# Low Threshold Microlasers Based on Organic-Conjugated Polymers

**DOI:** 10.3389/fchem.2021.807605

**Published:** 2021-12-13

**Authors:** Hong-xu Chen, Meng-dan Qian, Kun Yu, Yu-fang Liu

**Affiliations:** ^1^ Henan Key Laboratory of Infrared Materials and Spectrum Measures and Applications, School of Physics, Henan Normal University, Xinxiang, China; ^2^ School of Artificial Intelligence, Jilin University, Changchun, China

**Keywords:** organic-conjugated polymers, microlasers, resonator configurations, micro/nanofabrication techniques, optical sensing

## Abstract

Conjugated polymers have emerged as ideal organic laser materials for the excellent optoelectrical properties and facile processability. During a typical lasing process, resonator configurations with specific geometry are essential to provide optical feedback and then amplified light. Herein, we summarized the geometry and working mechanism of several typical resonator configurations formed with conjugated polymers. Meanwhile, recent advances in fabrication techniques and lasing performance are also discussed to provide new ideas for the design and optimization of microcavity geometries. Followed by the advances of practical applications in fields of laser sensing, bioimaging, and laser illumination/display, we make a summary of the existing bottlenecks and future perspectives of electrically driven organic lasers toward laser display and illumination.

## Introduction

In the past decades, lasers have led to great revolution in technology and industrial fields because of their unique and excellent properties, including high intensity, good monochromaticity, directionality, and strong coherence. The emergence of laser has greatly accelerated the development of technologies in industrial manufacturing, telecommunication, and biomedical science (Samuel and Turnbull, 2007). For instance, the scientific cognition is largely improved because of the ultra-high sensitivity and resolution in spectroscopy brought by lasers. Besides, lasers with low pumping thresholds hold promising applications in the display and lighting industry for their high brightness and monochromaticity that contribute to the highly vivid colors (Chellappan et al., 2010). The development of new lasers depends mainly on the advances of gain materials. According to early reports, both inorganic emitter materials and organic gain materials were well applied in the generation of lasers. The former materials, mostly inorganic semiconductors, generally have stable properties but inherent brittleness, requiring sophisticated processing techniques and costly configurations. By contrast, organic gain materials are easily available and mechanically flexible, allowing convenient fabrication of integratable photonic devices with low-cost processing equipment (Grivas and Pollnau, 2012). Moreover, organic molecules are of wide tunability in spectroscopy, and the chemical structures can be easily modified to satisfy the requirement of specific lasing wavelength and low pumping threshold (Xia et al., 2003). It has been found that various organic materials had high optical gain property and could be used as laser gain media, including some small organic dyes and conjugated polymers (Kuehne and Gather, 2016).

**FIGURE 1:**
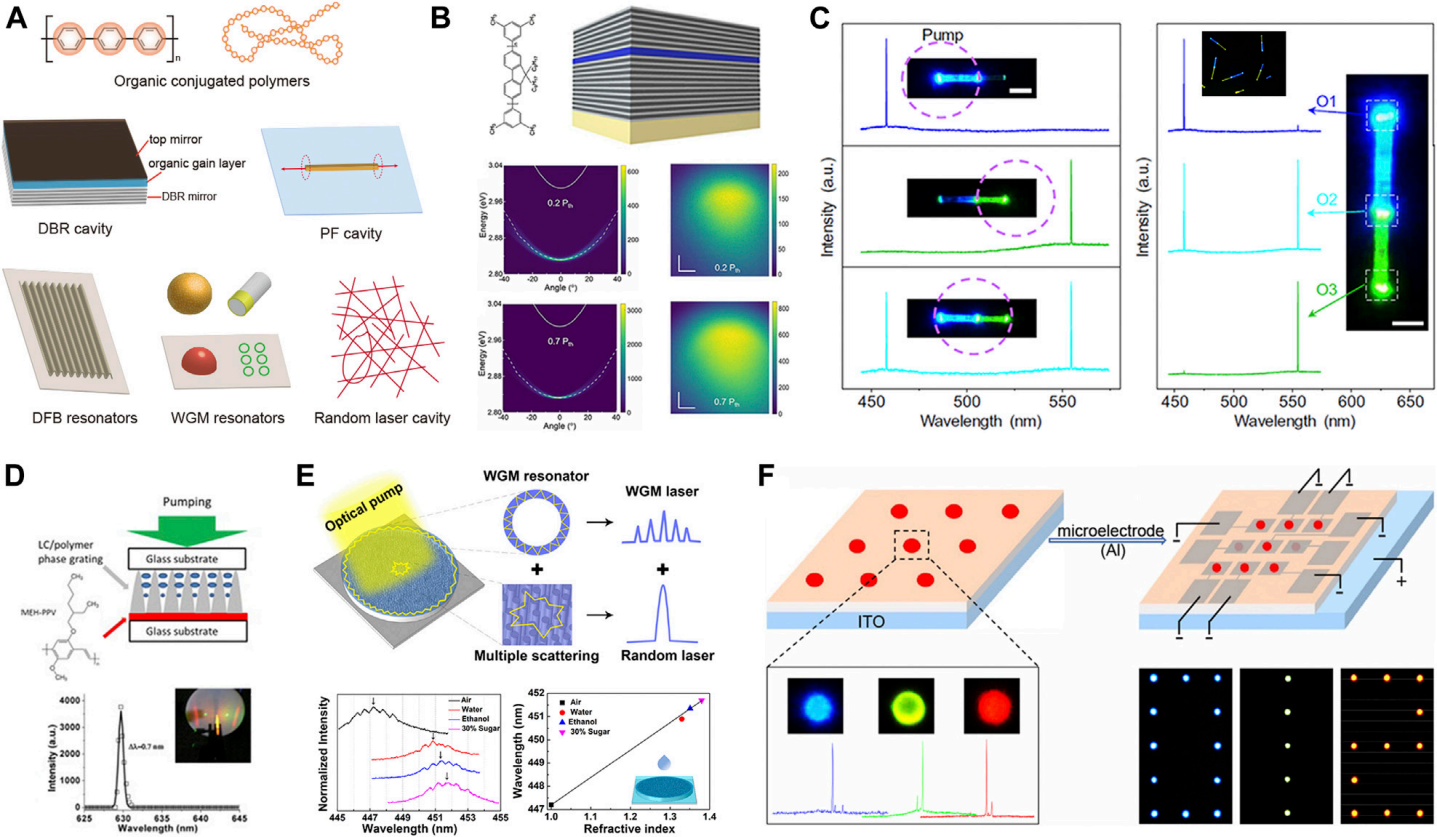
**(A)** Schematic framework of π-conjugated polymers and resonator configurations for organic laser, **(B)** A typical planar microcavity composed of poly(9,9-diochylfluorene (PFO) film and distributed Bragg reflector (DBR) mirrors for the generation of low threshold polariton lasers; reproduced with permission from Wei et al. (2019). **(C)** Axially coupled nanowire resonators for the modulation of laser modes based on the mutual mode mechanism; reproduced with permission from Zhang et al. (2017). **(D)** Conjugated polymer-associated distributed feedback (DFB) lasers prepared by the holographic polymerization method. **(E)** The whispering gallery mode (WGM) random laser from PFO/Ag-nanoparticle compound cavity used for potential sensing applications of different liquids; reproduced with permission from Xu et al. (2020). **(F)** Microscale light-emitting electrochemical cell arrays based on conjugated polymers and electrolytes for optical pumping laser and the potential electrically driven laser panel display; reproduced with permission from Liang et al. (2020).

Organic-conjugated polymer is an attractive gain material because of the excellent optoelectronic property and structural processability for device fabrication (Amarasinghe et al., 2009). Conjugated polymers usually comprise long chain units with alternate single and double C–C bonds, which are responsible for their semiconducting property (Figure 1A). The remarkable photoelectronic properties make conjugated polymers a good laser alternative for some inorganic materials (McGehee and Heeger, 2000). The first observation of organic laser from conjugated polymers was reported in 1992 with poly[2-methoxy, 5-(2′ethyl-hexoxy)-p-phenylenevinylene] (MEH-PPV) solutions as gain media (Moses, 1992). Then solid-state lasers based on semiconducting polymers attracted more attention and made remarkable advances for its practical prospects in integrated optoelectronic devices. Compared with some small organic laser molecules, conjugated polymers show unparalleled merits such as high absorption coefficients, broad spectra across the visible region, and most importantly, the resistance to light quenching at solid-state thanks to the long-chain barriers among each other, which make them an attractive low-threshold laser material for research (Xia et al., 2004). In addition to the optical source, some conjugated polymers can also be electrically pumped to emit bright fluorescence and laser (Rothe et al., 2006), which greatly broaden their practical foreground in optical sensing and electrically driven laser display.

In this mini review, we summarize the recent advances of conjugated polymers emitting low-threshold organic lasers with focus on the resonator configurations and patterning strategies involved, and then typical applications of organic microlasers in biological/chemical sensing and organic laser display are discussed. Finally, we provide the perspectives on the existing challenges and future outlooks of conjugated polymer-induced organic lasers.

## Optical feedback configurations and fabrication strategies

In a typical lasering process, pumping sources, gain materials, and resonator configurations are all essential to amplify light (Scherf et al., 2001). Under the proper excitation of light source, gain materials are stimulated to realize population inversion and the subsequent amplified oscillation by resonance. Then amplified emission of optical waves comes into being with the coherent optical field provided by resonator feedback configurations. By virtue of resonator configurations, organic lasers can be generated with low threshold and specific lasing mode. According to the optical feedback mechanism, resonator configurations for lasing action can be generally classified as distributed Bragg resonators (DBRs), Fabry–Pérot (FP) cavities, distributed feedback (DFB) resonators, whispering gallery mode (WGM) cavity, and random lasing configurations (Figure 1A) (Chénais and Forget, 2012). Availability of mature micro/nano-manufacturing techniques (Holdcroft, 2001), such as prepatterned template, micromolding, photolithography, interference-holographic lithography, electron beam lithography, and nanoimprinting, allows the diverse fabrication of functional resonator configurations, which significantly accelerate the development of organic lasers.

### Distributed Bragg resonators

The first introduction of microcavity lasers was realized by the group of Tessler, who fabricated a planar cavity consisting of poly (para-phenylene vinylene) (PPV) interlayer between two mirrors (Tessler et al., 1996). One of the mirrors has high reflectance (e.g., distributed Bragg reflector, DBR), and the other one is a metallic mirror that only allows partial transmission of light. The gain medium used for lasers only had a thickness of 100 nm, resulting in increasing gains and corresponding lower lasing threshold (∼100 nJ). This sandwich microcavity shows a sort of Fabry–Pérot resonance property where standing-wave optical field is supported between two mirrors. The DBR resonator axis is usually perpendicular to the plane of substrate film and mirrors. The planar resonator configuration can be prepared by simple spin coating or deposition of gain polymer laser on the DBR substrate. Chen et al. designed a two-dimensional DBR resonator consisting of four Bragg mirrors, which emitted nearly diffraction-limited polymer lasers with an ultralow threshold of 17 μJ/cm^2^ (Huang et al., 2016). Moreover, the size parameters of DBR geometry were investigated to be 40 μm to ensure an optimal working performance. Recently, polariton lasing with a low threshold of 27.7 μJ/cm^2^ was observed from disordered PFO film in a DBR cavity (Wei et al., 2019). This work broadens the scope of conjugated polymers in polariton laser devices with typical features of decreased linewidth, nonlinear emission intensity, and long-range spatial coherence (Figure 1B). Then the threshold of polariton lasing was further decreased to an ultra-low level (17 μJ/cm^2^) using pentafluorene as gain materials in a planar microcavity, where SiO_2_ and Ta_2_O_3_ layers were used for the construction of DBR microcavity, and the pentafluorene film was protected by a 10-nm buffer layer from degradation during the device fabrication process (Rajendran et al., 2019). The above work provides novel ideas for the fabrication of polaritonics.

### Fabry–Pérot cavities

Most of one-dimensional microscopic wires/fibers present typical Fabry–Pérot feedback features with the body part of wire showing good waveguiding capacity and the tip parts acting as mirrors (Matino et al., 2019). Conjugated polymers with high gain properties can be fabricated to micro/nanowires *via* facile techniques such as prepatterned templating, micromolding, and self-assembly. The typical fabrication work of conjugated polymer nanowires came from the Redmond group who used the porous alumina template assisted with the melt-mold method to prepare high yields of poly(9,9-dioctylfluorene) (PFO) nanowires (O'Carroll et al., 2007a). The nanowires generally exhibit regular morphology and good dispersity with homogeneous size ranging from 250 to 300 nm in diameter. Notably, the semicrystalline PFO nanowire presents excellent waveguiding performance, and the optically pumped microlaser at 460 nm is observed (O'Carroll et al., 2007b). They subsequently used a similar templating technique to obtain poly[(9,9-dioctylfluorenyl-2,7-diyl)-co-(bithiophene)] (F8T2) nanowires, which showed superior working performance in the field of photodetectors (O'Brien et al., 2006). As for some conjugated polymers with small repeat units, uniform crystal structures tend to be formed for the lack of interference from side chains.

The self-assembly method is also a good choice to fabricate a nanofiber of conjugated polymers in low molecular weight. It has been reported that oligo (p-phenylenevinylene) (OPV) derivatives, exhibiting extended π-conjugated system, are prone to form ordered nanowire crystals with lasing action. An axially coupled microcavity system was constructed from two kinds of nanowires assembled by distinct OPV derivatives. Zhao group proposed a mutual mode selection strategy using the coupled microwires to obtain multicolor single-mode lasing generation and alternative output modulation (Zhang et al., 2017). When pumped by a proper light, gain materials can radiate laser from the microwire cavity facet, which can be coupled into the other cavity, and thus, the microwires mutually act as laser source as well as wavelength filter cavity, finally resulting in the laser mode selection and modulation (Figure 1C). A similar self-assembly method is employed to readily fabricate microbelts of another OPV derivative. Combined with the femtosecond laser processing technique, the microbelt can be perfectly tailored into the microfiber array along its length direction. The microfiber units are capable of presenting a similar lasing action simultaneously (Liao et al., 2015). Modulation of multicolor laser mode and multipoint lasing action provide a more comprehensive idea of the utility of organic lasers, making the assembly of FP resonators as promising candidates in full-color laser display devices as well as integrated photonic circuits.

### Distributed feedback configurations

DFB geometries with diffractive periodic structures have emerged as a highly popular resonator configuration for its high optical feedback efficiency, which can efficiently avoid the large reflectivity caused by mirror or facet parts in FP cavity. When pumped by proper light, low-threshold lasering action will be generated from gain media under the interfering effect of periodic geometry (usually grating or photonic structures). According to the Bragg equation,

$$m\lambda_{L}\;=2n_{eff}\Lambda$$

where n_eff_ is the effective refractive index of the active layer, Λ is the grating period, and m is the Bragg order. Constructive interference only occurs on lasers with specific wavelengths (*λ*_L_).

Compared with other lasing systems, DFB configurations show special superiority in single-mode laser generation and broad-range spectral modulation by rational design of cavity parameters, such as grating period, refractive index, and thickness of gain layer (Heliotis et al., 2004; Klinkhammer et al., 2012), and the lasing spectra of conjugated polymers almost cover the whole visible region, thanks to the diverse material availability and flexible fabrication of grating parameters. A large amount of DFB laser work based on conjugated polymers have been reported during the past 20 years, mainly aiming to reduce the lasing threshold and improve the device fabrication techniques (Stehr et al., 2003).

The remarkable breakthrough on lasing threshold was achieved by the Bradley group, who intelligently proposed a mix-order (first and second order) grating strategy to reduce the lasing threshold of a polymer as low as 4 W/cm^2^ (Karnutsch et al., 2007). Subsequently, a combination of improved gain properties with developed DFB geometry further reduced the lasing threshold (0.77 kW/cm^2^) of conjugated polymers, making them possible to be pumped by a single LED source (Tsiminis et al., 2013). The primary DFB configuration consisted of grating templates as resonator and composite conjugated polymers as gain material. Convenient as it is, the lasing action is highly dependent on the grating substrate, which greatly limits the laser modulation capacity. In recent years, various micro/nanofabrication techniques have been employed to simplify the fabrication process and meanwhile increase the diversity and flexibility of grating configurations. The developed imprinting technique, as proposed by Prof. List, was subsequently employed to directly mold conjugated polymers into DFB gratings that presented desired lasing performance, which significantly improved the fabrication efficiency toward mass production (Gaal et al., 2003). Furthermore, advanced direct writing techniques including both electron-beam lithography (EBL), holographic polymerization technique, and laser interference ablation provided a more flexible way to achieve one-step and mass construction of one-dimensional or even complicated two-dimensional DFB lasing configurations (Kuehne et al., 2011; Zhai et al., 2011; Huang et al., 2012).

Distinct to optically driven organic lasers, electrically pumping lasers have attracted more and more attention because of its practicability and feasibility in the fields of organic laser diodes and full-color laser display. However, electrically driven laser is still confronted with serious challenges such as the extra additional loss and high lasing threshold. It has been validated that conjugated polymers with excellent charge transport capacity are ideally suitable for electrical pumping (Rothe et al., 2006). Besides, introduction of low-threshold DFB configurations into the electrically driven laser system can further reduce the pumping power intensity. Samuel et al. demonstrated an indirect electrically pumped laser configuration, in which InGaN LED was electrically driven as light source for the DFB lasing action of conjugated polymers (Yang et al., 2008). Subsequently, Heeger et al. constructed PPV-based 1D/2D grating resonators *via* nanoimprint technique to realize electrically pumped DFB laser at a low threshold (32 nJ/pulse) (Namdas et al., 2009). The electrical lasing threshold was further decreased as reported in the recent work. Oligo-(p-phenylene)-based gain materials with desirable carrier mobility were used in conjunction with DFB geometry, generating blue organic lasers at a very low threshold of only 0.16 nJ/pulse (Wei et al., 2017).

### Whispering gallery mode cavities

Whispering gallery mode (WGM) resonator is a kind of typical cavity that has good optical confinement capacity for the total internal reflection effect near the structural boundary. A variety of micro-configurations has been developed to generate WGM lasers, including micro-spheres/hemispheres, microbubbles, microdisks, microrings, and microcapillaries (Yang et al., 2015). These structures usually exist in more than one axis of symmetry so that light can be readily confined in the cavity, resulting in high-quality factors and low lasing thresholds. Various WGM lasers have been reported from conjugated polymers, among which microspheres are more popular due to the superior feedback property and facile fabrication techniques. For example, Xiao et al. demonstrated a tunable optofluidic microlaser from microbubble cavities. The smooth hollow bubble filled with polymer solutions enables the low lasing threshold of 7.8 μJ/cm^2^ (Tang et al., 2018). In comparison with liquid laser cavities, solid ones possess unique advantages in the miniaturization and integration of organic laser devices. Xu et al. have proposed a novel WGM random cavity to achieve double-mode lasing simultaneously (Figure 1E). By virtue of inkjet printing and etching method, the size of cavity can be tailored flexibly to modulate the lasing mode, which shows promising prospects in chemical sensing (Xu et al., 2020). Besides, conjugated polymers self-assemble to well-defined solid microspheres using the simple vapor diffusion precipitation method, successfully reducing lasing threshold as low as 0.37 μJ/cm^2^ (Kushida et al., 2017). Very recently, Zhao et al. remarkably constructed a light-emitting electro-chemical cell micro-array consisting of conjugated polymers and electrolytes (Figure 1F). Every single WGM unit acted as a lasing pixel under proper optical excitation, and meanwhile, electrical driven luminance was also realized. Even though the electrical pumping laser did not realize for the additional loss in this configuration, it provides novel perspectives for the construction of electrical driven laser display devices (Liang et al., 2020).

### Random lasing cavities

Conjugated polymers without specific feedback resonators can also present a lasing action, called random laser, when multiple scattering occurs in the irregular region. Random laser usually appears from gain medium with ill-defined and unordered geometries. Nanowires based on a typical π-conjugated oligophenyl material have been synthesized by the simple hot wall epitaxy method, and low-threshold random lasing action of nanowires (0.5 μJ/cm^2^) was measured at ∼425 nm under optical pumping (Quochi et al., 2005). Bongiovanni et al. further investigated the temperature-dependent nonlinear exciton process of the random lasing action from these nanofibers (Quochi et al., 2008). The facile fabrication techniques and high intensity of random laser enable its promising applications in sensing and optical imaging. Li et al. have taken advantage of random lasing from TiO_2_ nanoparticle-doped TPA-PPV films to detect explosive vapors. The sensitivity of random laser was enhanced more than 20 times than that in spontaneous emission (Deng et al., 2010).

## Applications

As an excellent laser material, conjugated polymers in proper resonator configurations can be pumped optically or electrically to generate intense organic lasers, which is expected to play important roles in diverse practical applications, such as chemosensing, biological sensing and imaging, organic laser diode, and electrically driven multicolor laser display (Toropov et al., 2021). Microlasers with high quality factor are highly suitable for optical sensing since tiny variations of the surrounding environment and material properties will induce drastic changes in lasing action. Explosive vapors are important analytes for conjugated polymers since their luminescent emission can be effectively quenched by explosive molecules (Yang et al., 2010). By virtue of this mechanism, Bulović et al. originally utilized the lasing behavior rather than spontaneous emission of PFO to detect explosive analytes, and the sensitivity enhanced more than 30 times. Introduction of DFB, WGM, and random lasing mode resonators allows further enhancement of sensitivity, achieving ultra-low detecting vapor pressure of 9.8 and 5 ppb for DNB and TNT, respectively (Rose et al., 2005). Xu et al. put forward a compound cavity containing PFO and Ag nanoparticles to achieve WGM random lasers, which vary in wavelength and intensity under stimuli of different liquids (Figure 1E) (Xu et al., 2020). This novel configuration has promising applications in laser sensing. In terms of biological applications, a DFB laser configuration combining conjugated polymers and hydrogel recognition layer has been fabricated to realize label-free and real-time measurement of avidin–biotin interaction (Heydari et al., 2014). Recently, Kuehne et al. have synthesized conjugated polymer-based microparticles, which emit both WGM lasers and Raman scattering narrow-band emission. By detecting the lasing and Raman scattering signals simultaneously, cells phagocytizing microparticles are well tracked and imaged with reduced interferences (Haehnle et al., 2020). Apart from sensing applications, electrically driven laser action also plays an important role in laser diodes and panel display, accelerating the development of integrated luminescent devices with low-cost and flexible features (Liang et al., 2020).

## Conclusion and outlook

In this minireview, we have focused on the organic lasing action and practical applications of conjugated polymers. Optical feedback configurations and relevant micro/nano-fabrication strategies are well summarized to provide an overall perspective on recent developments and existing problems toward polymer lasers. Even though considerable efforts have been made to simplify the fabrication techniques for resonator configurations and further reduce the lasing threshold, there still exist many bottlenecks urgently to be solved. First, it is expected that integratable lasing resonators in micro–nano scale can be further developed to ensure their practical applications in integrated laser source and full-color laser display. Besides, as a very appealing but intractable issue, electrically driven organic laser has not been totally realized in conjugated polymers due to the low carrier density and high additional losses associated with electrode absorption, polaron quenching, and triplet absorption. Even though diverse approaches focusing on the abovementioned problems have been attempted based on tailored gain material with increased carrier mobility, further efforts should be put on the optimization of resonator configurations to reduce the additional losses and achieve the electrically operated organic lasers with low threshold.
